# New Therapeutic Method for Alleviating Damage of Acute Kidney Injury Through *BCL-2* Gene Promoter I-Motif

**DOI:** 10.3390/ijms252212028

**Published:** 2024-11-08

**Authors:** Dongsheng Ji, Jiahui Zhang, Jihai Liang, Jing Wang, Xiaoya Li, Zhi-Shu Huang, Ding Li

**Affiliations:** School of Pharmaceutical Sciences, Sun Yat-sen University, Guangzhou University City, Guangzhou 510006, China; jidsh@mail2.sysu.edu.cn (D.J.); zhangjh265@mail2.sysu.edu.cn (J.Z.); liangjh225@mail2.sysu.edu.cn (J.L.); wangj349@mail2.sysu.edu.cn (J.W.); lixiaoya0908@126.com (X.L.); ceshzs@mail.sysu.edu.cn (Z.-S.H.)

**Keywords:** acute kidney injury, anti-apoptosis, BCL-2, i-motif, transcriptional regulation, acridone derivative

## Abstract

Acute kidney injury (AKI) is a global public health problem with its pathogenesis not fully understood. Excessive apoptosis of renal tubular epithelial cells is an important feature of AKI patients, and therefore an anti-apoptotic approach could be used in the treatment for AKI. Up-regulation of B-cell lymphoma-2 (*BCL-2*) gene and protein has been found to be correlated with anti-apoptosis of cells. It has been found that the presence of the C-rich sequence on the upstream region of the *BCL-2* gene promoter could form DNA secondary i-motif structure, and its stabilization by small molecules could up-regulate gene transcription and translation. In the present study, we constructed AKI models through folic acid (FA) induction. With these in vitro and in vivo models, we demonstrated that the acridone derivative **A22** could up-regulate the expression of BCL-2 by targeting its gene promoter i-motif to reduce renal tubular epithelial cell apoptosis and improve renal function in many ways. **A22** could alleviate FA-induced oxidative stress injury, inflammatory response, and endoplasmic reticulum stress in mouse kidneys. Our results provided a potentially new anti-apoptotic approach for the treatment of early stages of AKI. Our employed model focused on its short-term effect on AKI, while its long-term efficacy and safety, particularly regarding the regeneration of renal tubular epithelial cells, require further investigation before clinical application. This study further demonstrated that promoter i-motif could be targeted for up-regulating BCL-2 expression for the treatment of important diseases caused by excessive apoptosis.

## 1. Introduction

Acute kidney injury (AKI) is a complex syndrome that occurs within a few hours or days and is characterized by reduced renal function, with high incidence and mortality [1,2,3]. The etiology of AKI is a consequence of prerenal, intrarenal, and postrenal factors [4]. Prerenal factors are associated with decreased glomerular filtration rates due to decreased renal perfusion pressure as an adaptive response to external injury, with sepsis [5] as a common trigger. The intrarenal factor involves the tubular, glomerular, interstitial, and vascular systems, usually associated with ischemic reperfusion [6] and nephrotoxic drugs [7]. Postrenal factor is associated with urinary tract obstruction due to factors such as tumors and stones. COVID-19 has been reported to be closely associated with AKI [8,9], and in addition, studies have found that inflammation [10], oxidative stress [9], and renal tubular epithelial cell apoptosis [11] play important roles in the development and progression of AKI. The etiology and pathogenesis of AKI are complex, so an accurate diagnosis of the underlying etiology is the key to successful treatment, and the current clinical approach focuses on supportive therapy and renal replacement therapy. General management principles for AKI include determination of volume status, fluid resuscitation with isotonic crystalloid, treatment of volume overload with diuretics, discontinuation of nephrotoxic medications, and adjustment of prescribed drugs according to renal function. Additional supportive care measures may include optimizing nutritional status and glycemic control. The pathomechanism of AKI is not fully understood, and no specific pharmacologically targeted drug is available for the treatment of AKI. Therefore, effective targeted therapy is urgently required.

The pathogenesis of AKI is multifactorial, and relevant studies have confirmed that patients’ renal tubular epithelial cell apoptosis could lead to a rapid decline in renal function in a short time [11]. It is noteworthy that it has also been suggested that moderate apoptosis is a necessary mechanism for the clearance of damaged cells and can promote renal repair [12]. The BCL-2 family could regulate the checkpoint of apoptosis through anti-apoptotic subfamily proteins such as BCL-2 and pro-apoptotic subfamily proteins such as BCL2-associated X protein (BAX). The expression of anti-apoptotic BCL-2 has been shown to be reduced in injured kidneys [13]. Therefore, we hypothesized that restoration of BCL-2 expression levels could be important for the anti-apoptosis of renal tubular epithelial cells for AKI. It has been shown that up-regulation of *BCL-2* gene transcription and translation could be achieved through the stabilization of its promoter i-motif structure [14,15], which have also been demonstrated for other gene promoter i-motifs [16]. The i-motif structures are formed by two parallel-stranded C:C^+^ hemiprotonated base-paired duplexes intercalating in an antiparallel manner [17], mostly under acidic conditions [18] or molecular crowding conditions in vitro [19]. It has been demonstrated that the i-motif could be present in a variety of DNA structural regions, such as telomeres [20,21,22,23], proto-oncogene promoter regions [24], and centromeres [25,26]. It has been shown that the promoter and telomeric regions have significant i-motif structures by using the i-motif-specific antibody i-Mab [20]. In our previous work, we found that acridone derivative **B19** could selectively down-regulate c-myc gene transcription and translation by targeting c-myc promoter i-motif [27].

In a previous study, we discovered that acridone derivative **A22** could up-regulate the transcription and translation of BCL-2 by binding to and stabilizing *BCL-2* promoter i-motif structure, which could alleviate the hepatic apoptosis-associated pathological features of nonalcoholic fatty liver disease (NAFLD)/nonalcoholic steatohepatitis (NASH) in mouse models [28]. This anti-apoptotic approach provides a novel therapeutic strategy for diseases resulting from excessive apoptosis, which prompted us to further the application of **A22** in other models of diseases caused by excessive apoptosis. In this study, we assessed the therapeutic effect of **A22** on apoptosis-associated pathological features in vitro and in FA-induced AKI mouse models. Our knockdown experiment confirmed that **A22** functioned by binding to and stabilizing *BCL-2* promoter i-motif. **A22** showed a significant therapeutic effect on the AKI model by lessening renal tubule epithelial cell apoptosis, possibly through up-regulating BCL-2. **A22** showed a significant effect on the expressions of apoptosis initiation and execution factors, including cleaved caspases 3 and 9, kidney injury in relation to serum creatinine and blood urea nitrogen, and renal inflammatory response in relation to the number of macrophages.

Although gene promoters G-quadruplex and i-motif have been utilized as potential targets and widely studied for cancer treatment, these special secondary structures have seldom been applied for studies of diseases besides oncology with small molecule binding ligands. To our knowledge, this is the first attempt to use gene promoter i-motif as a therapeutic target for AKI with a small molecule. Compared with other technologies for upregulating gene expression, small molecule binding ligands have general advantages of economy, simplicity, and realizing and preserving drugs easily, which provides a new strategy for drug research and development against AKI.

## 2. Results

### 2.1. **A22** Showed Dose-Dependent Anti-Apoptosis in FA-Induced Apoptosis Cell Model

**Figure 1:**
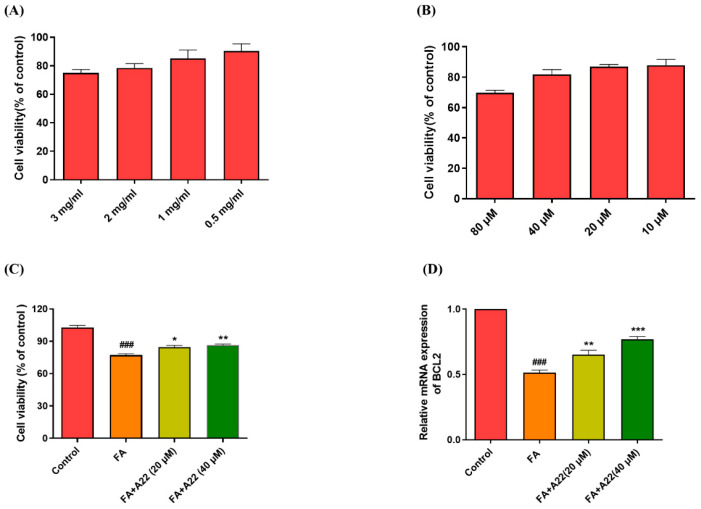

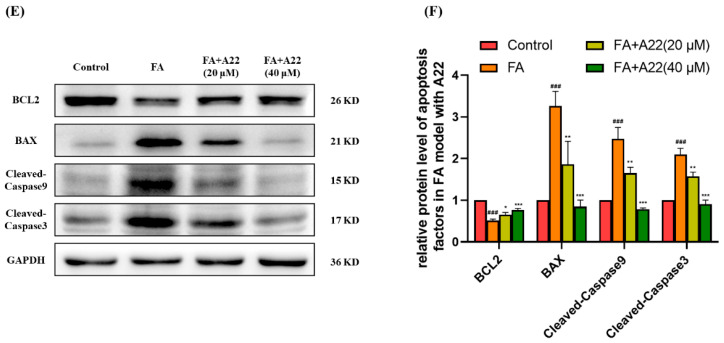
Effect of **A22** on anti-apoptosis in a 3 mg/mL folic acid (FA)-induced cell model. (**A**) Cellular viability of HK-2 cells induced by FA at different doses. (**B**) Cellular viability of HK-2 cells with various concentrations of **A22**. (**C**) The effect of **A22** on the viability of HK-2 cells induced by FA was measured by using the cell counting kit-8 (CCK-8) assay upon treatment of the cells with increasing concentrations of **A22**. (**D**) The mRNA levels of BCL-2. (**E**) Effect of **A22** on expressions of apoptosis-related proteins in FA-induced HK-2 cells and (**F**) corresponding histogram. The extracted proteins from the HK-2 cells were immunoblotted with specific antibodies and quantified based on the loading control of GAPDH. Data were statistically analyzed as means ± SEM, with each circle or column indicating one group (N ≥ 3). ### *p* < 0.001, vs. Control group; * *p* < 0.05, ** *p* < 0.01, and *** *p* < 0.001 vs. FA group.

Excessive renal tubular epithelial cell apoptosis has been shown to be related to AKI [29], and therefore, inhibition of renal tubular epithelial cell apoptosis could block the course of AKI. Since the acridone derivative **A22** could up-regulate the expression of BCL-2 and alleviate the hepatic apoptosis-associated pathological features of NAFLD/NASH, in the present study, we further examined its anti-apoptotic effect on the FA-induced kidney cell apoptosis model. As shown in Figure 1A, after treatment with 3 mg/mL FA for 24 h, the cell survival ratio was reduced to 68%, which was used as a cell apoptosis model. Cell survival ratio was improved by 12% (*p <* 0.05) and 21% (*p* < 0.01) upon the addition of 20 μM and 40 μM **A22**, as shown in Figure 1C. We examined the levels of BCL-2 mRNA and protein in FA-induced models, with the results as shown in Figure 1D and Table S2. The mRNA level of anti-apoptosis-related factor BCL-2 was found to be reduced (*p* < 0.001) in the FA model, which was increased by 20% (*p <* 0.01) and 33% (*p <* 0.01) upon the addition of 20 μM and 40 μM **A22**. Additionally, the BCL-2 protein level was also found to be increased after **A22** administration compared with the FA group (Figure 1E). Then, we investigated the changes in protein expressions related to the apoptotic signaling pathway in FA-induced models. We found that **A22** could significantly reduce the protein expressions of apoptosis-related factors in FA-induced AKI models in dose-dependent manners, with reduced activation of cleaved caspases 3 and 9 (Figure 1E).

### 2.2. **A22** Showed Dose-Dependent Anti-Apoptosis in FA-Induced Apoptosis Cell Model with Significant Effect on Mitochondrial Number and Function

**Figure 2:**
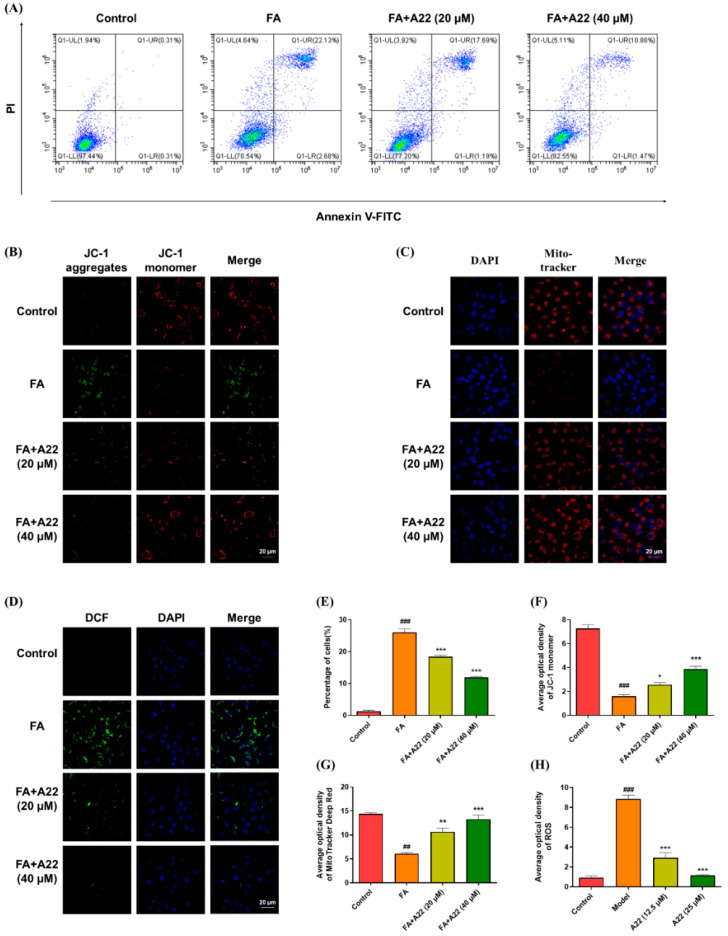
**A22** showed dose-dependent anti-apoptosis in a folic acid (FA)-induced apoptosis cell model without significant effect on mitochondrial number and function. (**A**) Flow cytometry analysis and (**E**) corresponding histogram. (**B**) Confocal images for the 5,5′,6,6′-tetrachloro-1,1′,3,3′-tetraethyl-imidacarbocyanine iodide (JC-1) fluorescent probe in HK-2 cells under different conditions and (**F**) percentage of cells. (**C**) Confocal images for the mitochondria of HK-2 cells under different conditions and (**G**) percentage of positive areas. (**D**) Confocal fluorescent images of intracellular reactive oxygen species (ROS) levels under different treatments and (**H**) percentage of positive areas. Cell nuclei were stained with 4′,6-diamidino-2-phenylindole (DAPI) dye (blue). ROS was marked with oxidizing non-fluorescent 2′,7′-dichlorodihydrofluorescein diacetate (DCF, green). The change in mitochondrial membrane potential was marked with JC-1 (red), and the number of mitochondria was marked with the Mito tracker deep red probe (red). Magnification was 60×, and the scale bar was 20 μm. Data were statistically analyzed as means ± SEM, with each circle or column indicating one group (N ≥ 3). ## *p* < 0.01, vs. Control group; ### *p* < 0.001, vs. Control group; * *p* < 0.05, vs. FA group; ** *p* < 0.01, vs. FA group; *** *p* < 0.001, vs. FA group.

The FITC Annexin V/PI apoptosis detection method was used for the collected HK-2 cells, which were analyzed by using flow cytometry on the FA-induced model group without or with different concentrations of **A22** treatment and control group. As shown in Figure 2A, in contrast to the control cells with an apoptotic ratio of less than 1%, after treatment with 3 mg/mL FA, the number of cells in the late stage of apoptosis increased to 24.81% (*p* < 0.001) of total cell number. Upon addition of 20 μM and 40 μM **A22**, the apoptosis ratio was reduced to 18.88% and 12.35% (*p* < 0.001), respectively, in a dose-dependent manner, indicating that **A22** had apparent anti-apoptotic activity. During the early stage of apoptosis, the membrane potential of mitochondria is changed, and the 5,5′,6,6′-tetrachloro-1,1′,3,3′-tetraethyl-imidacarbocyanine iodide (JC-1) fluorescent probe is widely used for the detection of early apoptosis due to its high sensitivity and membrane permeability to accurately indicate this change. Mitochondrial depolarization leads to a decrease in mitochondrial membrane potential in early apoptosis, as evidenced by a significant decline in the red/green fluorescence intensity ratio. After **A22** administration, the red/green fluorescence intensity ratio was increased, suggesting its protective effect on early apoptosis. Besides, the change in the level of reactive oxygen species and the number of mitochondria in cells before and after **A22** administration were studied by using the 2′,7′-dichlorodihydrofluorescein diacetate (DCF) probe and the Mito tracker deep red probe, respectively. We found that **A22** could alleviate oxidative stress with a significant effect on the mitochondrial number in living cells of the 3 mg/mL FA-induced apoptosis model, as shown in Figure 2. The results showed that **A22** could also alleviate apoptosis and enhance cell survival by reducing oxidative stress while maintaining mitochondrial number and function in the FA-induced apoptosis model.

### 2.3. **A22** Had Its Anti-Apoptotic Effect, Possibly Through Upregulation of BCL-2 by Binding to Its Gene Promoter I-Motif

It has been shown that **A22** can increase the binding of *BCL-2* promoter i-motif to transcriptional factor heterogeneous nuclear ribonucleoprotein L-like (hnRNP LL), which forms a complex to activate *BCL-2* gene expression [14,30]. In the present study, we reduced the expression of hnRNP LL in HK-2 cells by using siRNA technology, and then the expression of BCL-2 and mechanism for the anti-apoptotic effect of **A22** were investigated. As shown in Figure S1 and Table S2, after silencing the gene of hnRNP LL with siRNA technology, the mRNA level of hnRNP LL decreased by 65% compared with the control. Western blot was then used to analyze the change in protein expressions. Expression of hnRNP LL protein in the silent siRNA group decreased, indicating that siRNA transfection technology down-regulated the expression of the hnRNP LL gene. In the meantime, the expression of BCL-2 was significantly lower than the control group, indicating that hnRNP LL plays an important role in regulating the expression of BCL-2.

**Figure 3:**
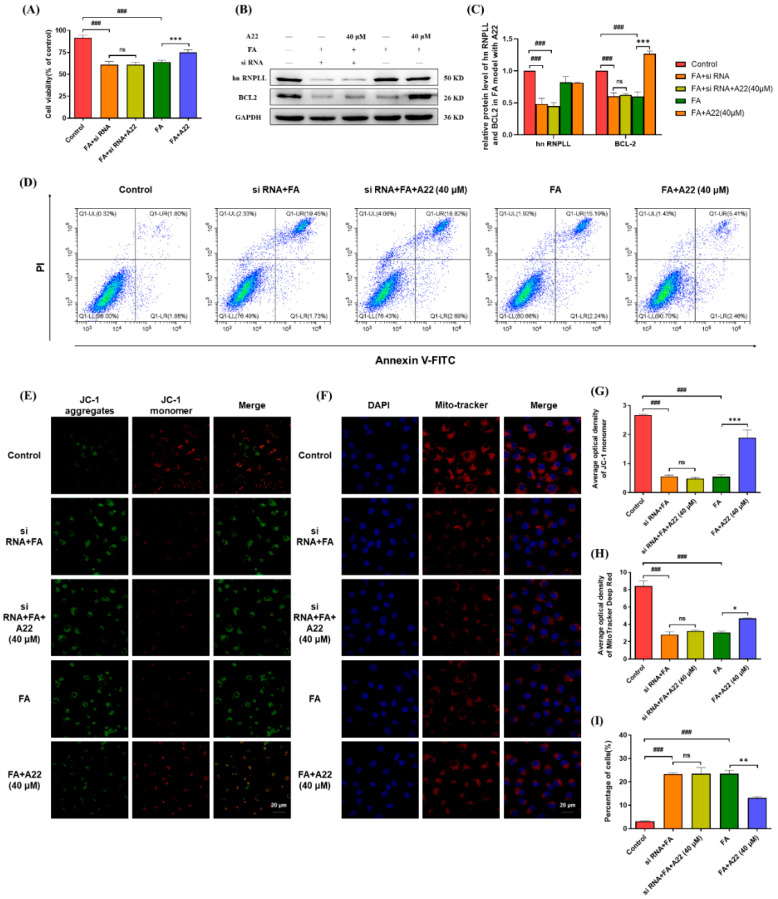
The anti-apoptotic effect of **A22** was found to be related to its up-regulation of BCL-2 studied in a folic acid (FA)-induced cell model before and after heterogeneous nuclear ribonucleoprotein L-like (hnRNP LL) knockdown. (**A**) HK-2 cell viability was measured in the presence and absence of **A22** before and after hnRNP LL knockdown. (**B**) The protein levels of BCL-2 were analyzed by using Western blot. (**C**) The mRNA levels of hnRNP LL and BCL-2 were analyzed. (**E**,**F**) Confocal images of the 5′,6,6′-tetrachloro-1,1′,3,3′-tetraethyl-imidacarbocyanine iodide (JC-1) fluorescent probe and mitochondria in HK-2 cells were taken under different conditions with the percentage of positive areas as shown in (**G**,**H**). (**D**) Flow cytometry analysis and (**I**) corresponding histogram. Magnification was 60×, and the scale bar was 20 μm. Data were statistically analyzed as means ± SEM, with each circle or column indicating one group (N ≥ 3). ### *p* < 0.001, vs. Control group; * *p* < 0.05, vs. FA group; ** *p* < 0.01, vs. FA group; *** *p* < 0.001, vs. FA group.

Then, this FA-induced AKI cell model with suppression of hnRNP LL gene expression using siRNA technology was used as a knockout model for investigating the anti-apoptotic effect of **A22**. As shown in Figure 3A, the survival ratio of the FA-induced knockout model was 63%, which was not changed upon incubation with 40 μM **A22**. In comparison, the cell survival ratio was increased by 16% upon incubation with 40 μM **A22** for the FA-induced normal model without suppression of hnRNP LL gene expression. We also comparatively studied the effect of **A22** on BCL-2 protein expression in the knockout model and normal model through Western blot. As shown in Figure 3B, the protein expression level of BCL-2 in the FA-induced knockout model decreased, which was not changed upon incubation with 40 μM **A22**. In contrast, the expression of BCL-2 increased (*p* < 0.001) upon incubation with 40 μM **A22** for the FA-induced normal model.

Flow cytometry was also used to comparatively study the anti-apoptotic effect of **A22** in knockout and normal models of HK-2 cells. As shown in Figure 3D, in contrast to the control cells with an apoptotic ratio of less than 2%, apoptotic cells in FA-induced knockout and normal model group were significantly increased to 21.18% (*p* < 0.001) and 17.43% (*p* < 0.001), respectively. Upon addition of 40 μM **A22**, apoptotic cells of the normal model group were reduced to 7.87% (*p* < 0.001), while apoptotic cells of the knockout model group were reduced to 19.51% (*p* < 0.001) only. The anti-apoptotic effect of **A22** in knockout and normal models of HK-2 cells was also studied with a JC-1 fluorescence probe (Figure 3E) and a Mito tracker deep red probe (Figure 3F). Upon the addition of 40 μM **A22**, the depolarization of mitochondria and the level of reactive oxygen species, as well as the number of mitochondria in cells, were not improved in the FA-induced knockout group. In comparison, in the normal group, **A22** showed a significant anti-apoptotic effect by alleviating mitochondrial depolarization and maintaining the number of mitochondria. These results indicated that **A22** up-regulated BCL-2 transcription and translation by binding to *BCL-2* promoter i-motif in the FA-induced AKI cell model, thus playing an anti-apoptotic role.

### 2.4. **A22** Could Improve Renal Function and Attenuate Morphological Injury of Kidney Following FA Administration in Mice

**Figure 4:**
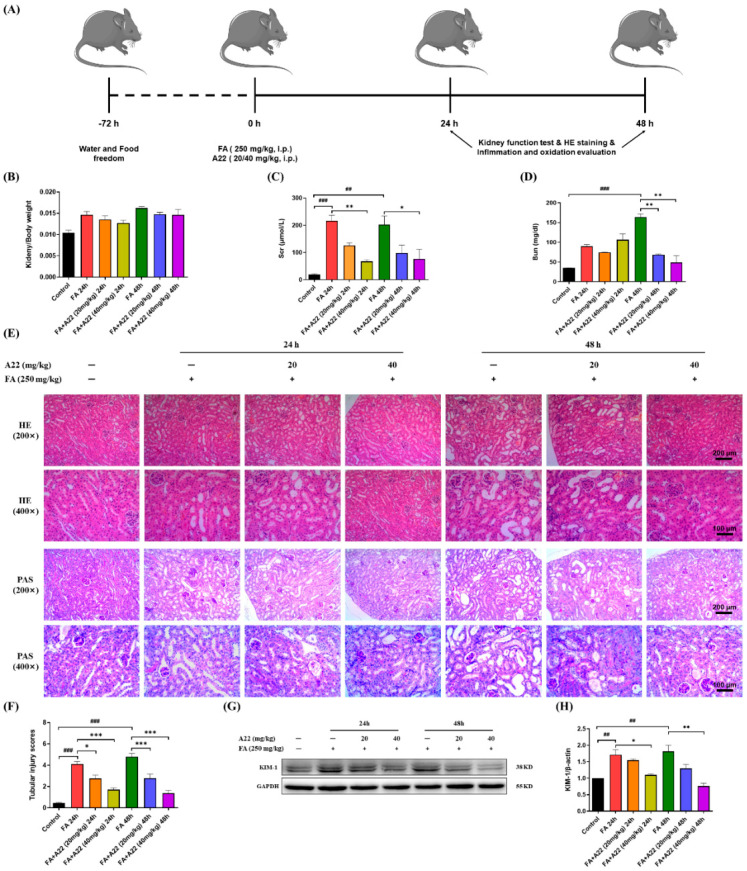
The effect of **A22** on the acute kidney injury (AKI) mouse model was studied with various body parameters determined. (**A**) Preparation and treatment schedule of AKI mice. (**B**) The data on kidney/body weight were determined under various conditions. (**C**) The data on serum creatinine (Scr) were determined under various conditions. (**D**) The data on blood urea nitrogen (Bun) were determined under various conditions. (**E**) Hematoxylin eosin (HE) and periodic acid-schiff (PAS) stain images of renal tissues under various conditions (magnification was 200×, and scale bar was 200 μm; Magnification was 400×, and scale bar was 100 μm). (**F**) Tubular injury scores were determined under various conditions. (**G**) The expressions of kidney injury molecule-1 (KIM-1) under various conditions and (**H**) corresponding histograms were determined. Data were statistically analyzed as means ± SEM, with each circle or column indicating one group (N ≥ 3 mice/group). ## *p* < 0.01, vs. Control group; ### *p* < 0.001, vs. Control group; * *p* < 0.05, vs. FA 24/48 h group; ** *p* < 0.01, vs. FA 24/48 h group; *** *p* < 0.001, vs. FA 24/48 h group.

To further evaluate the anti-apoptotic effect of **A22**, we carried out in vivo experiments. The AKI mouse model was constructed through FA induction, which was prepared and used following previously reported methods [31]. The results were obtained, as shown in Figure 4A, to evaluate the potential therapeutic effect of **A22** on apoptosis. The relevant studies were carried out after 24/48 h intraperitoneal injection of increasing concentrations of **A22**. The ratio of kidney weight vs. body weight (KW/BW) was found to be only slightly decreased upon treatment with **A22**. Serum creatinine (Scr) and blood urea nitrogen (Bun) are common indicators reflecting renal function in the study of AKI. As shown in Figure 4C,D, the blood biochemical analysis revealed an obvious reduction in the level of Scr and Bun in **A22**-treated AKI mice as compared to FA-induced mice. Hematoxylin–eosin (HE) and periodic acid-schiff (PAS) analyses of renal tissues were also performed. In FA-induced 24/48 h groups, HE staining showed that severe glomerular basement membrane (GBM) was damaged and renal tubules dilated obviously, with significant cell apoptosis occurring in some renal tubules. Similarly, PAS staining showed that casts appeared in renal tubules 24/48 h after FA induction. After 24/48 h injection of increasing concentrations of **A22**, as expected, cell morphology improved obviously, the number of apoptotic cells and casts decreased significantly, and renal damage was repaired. Meanwhile, the degree of renal tubular injury in HE staining was scored by using the Paller method, as shown in Figure 4F, and injury scores were significantly increased (*p* < 0.001, *p* < 0.001) in FA-induced 24/48 h groups. After injection of **A22**, injury scores decreased in dose-dependent manners. Kidney injury molecule-1 (KIM-1) is an important indicator of early or acute kidney injury, and FA could increase the level of KIM-1, as shown in Figure 4G,H. Compared to FA-induced 24/48 h groups, **A22** could significantly decrease the expression of KIM-1 (*p* < 0.05, *p* < 0.01) in dose-dependent manners. In addition, Masson’s Trichrome staining (Figure S2) showed that **A22** had a certain improvement in FA-induced early renal fibrosis. These results showed that **A22** could improve renal function and attenuate morphological injury of the kidney.

### 2.5. **A22** Could Up-regulate BCL-2 Expression and Ameliorate Apoptosis of Renal Tubule Cells in FA-Induced Mice Model

**Figure 5:**
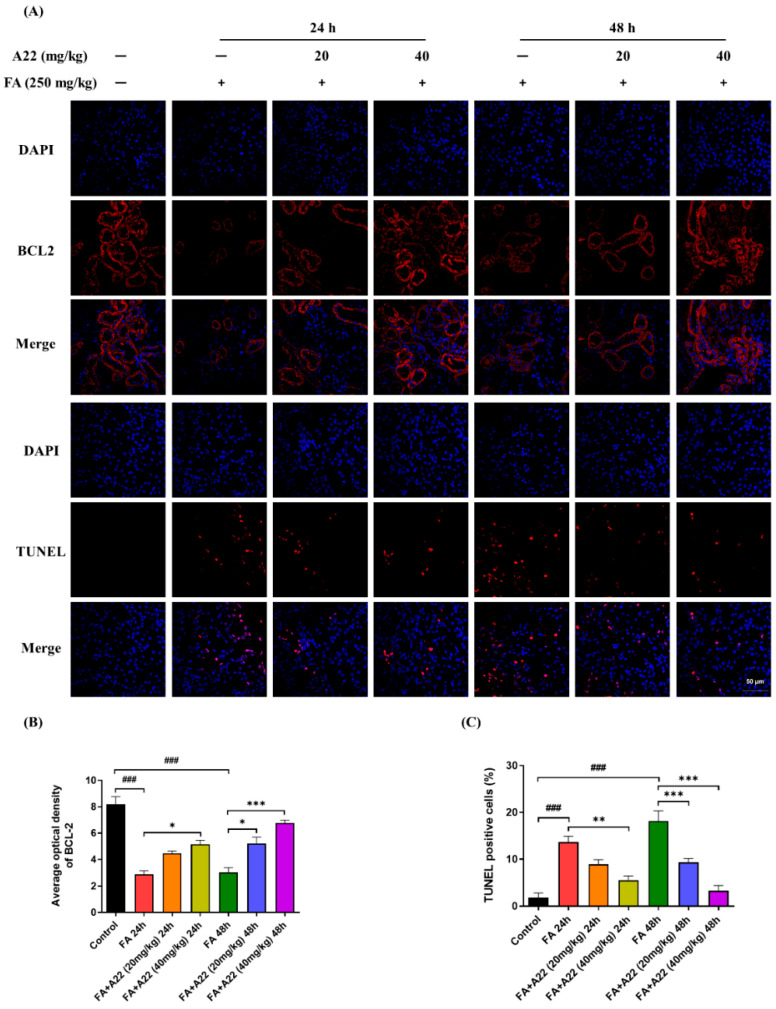
The result of the immunohistochemical stain of BCL-2 and terminal deoxynucleotidyl transferase-mediated deoxyuridine triphosphate nick-end labeling (TUNEL) experiments in mice model. (**A**) Fluorescence images of renal tissues were collected from different groups with immunohistochemical stains of BCL-2 and TUNEL assay, as well as corresponding histograms as shown in (**B**,**C**). Magnification was 60×, and the scale bar was 20 μm. DAPI was indicated with blue fluorescence, BCL-2 was indicated with red fluorescence, and TUNEL was indicated with red fluorescence. Data were statistically analyzed as means ± SEM, with each circle or column indicating one group (N ≥ 3 mice/group). ### *p* < 0.001, vs. Control group; * *p* < 0.05, vs. FA 24/48 h group; ** *p* < 0.01, vs. FA 24/48 h group; *** *p* < 0.001, vs. FA 24/48 h group.

Our above experiments showed that **A22** could ameliorate almost all pathological injuries in the FA-induced AKI mice model; therefore, further immunohistochemical and terminal deoxynucleotidyl transferase-mediated deoxyuridine triphosphate nick-end labeling (TUNEL) experiments were performed with results as shown in Figure 5A–C. After induction with FA for 24/48 h, the expression levels of BCL-2 in mouse kidneys were significantly decreased, which were then increased in dose-dependent manners upon treatment with **A22** (*p* < 0.05, *p* < 0.001). In the meantime, tunnel-positive cells in mouse kidneys were greatly increased after induction with FA for 24/48 h, which were then decreased in dose-dependent manners upon treatment with **A22** (*p* < 0.01, *p* < 0.001).

**Figure 6:**
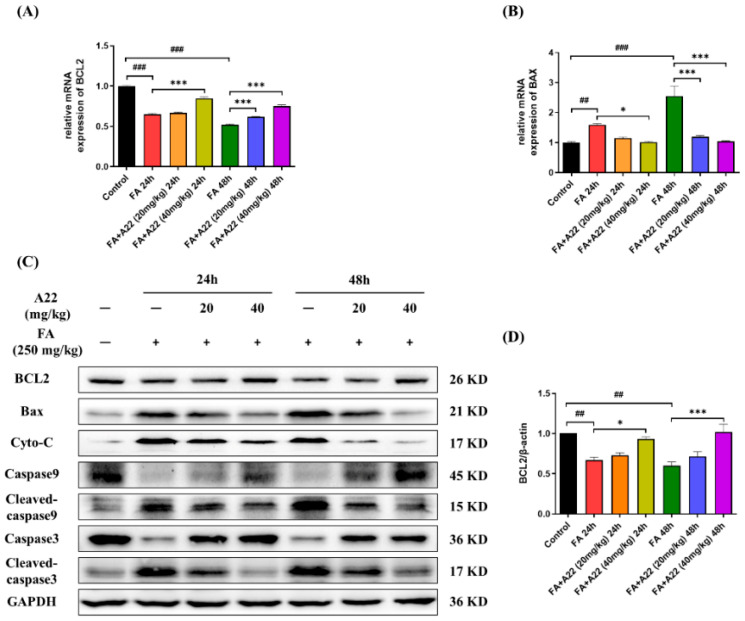

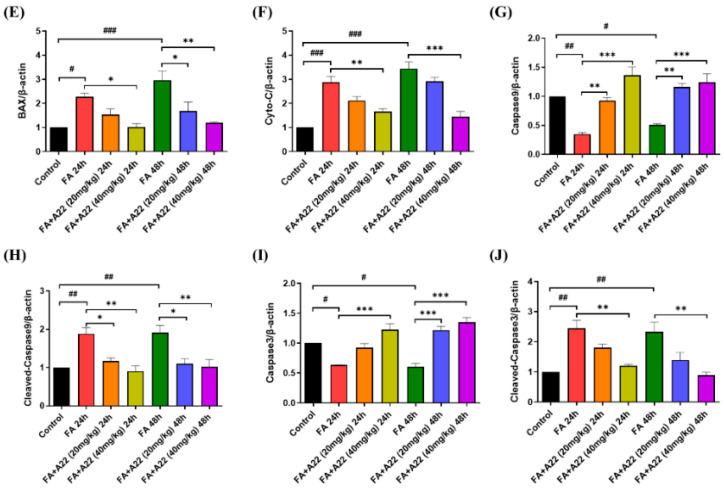
Effect of **A22** on ameliorating apoptosis in folic acid (FA)-induced mice model. (**A**) Effect of **A22** on *BCL-2* gene transcription. (**B**) Effect of **A22** on BAX gene transcription. (**C**) Effect of **A22** on expressions of apoptosis-related proteins in kidney and (**D**–**J**) corresponding histograms. The extracted proteins from the kidney were immunoblotted with specific antibodies and quantified based on the loading control of GAPDH. Data were statistically analyzed as means ± SEM, with each circle or column indicating one group (N ≥ 3 mice/group). # *p* < 0.05, vs. Control group; ## *p* < 0.01, vs. Control group; ### *p* < 0.001, vs. Control group; * *p* < 0.05, vs. FA 24/48 h group; ** *p* < 0.01, vs. FA 24/48 h group; *** *p* < 0.001, vs. FA 24/48 h group.

To further confirm the anti-apoptotic effect of **A22**, we examined the transcription and translation of apoptosis-related factors in mouse kidney samples. As shown in Figure 6A,B and Table S2, the transcriptions of BCL-2 and BAX had significant changes in FA-induced AKI models, with their mRNA levels analyzed by using quantitative real-time polymerase chain reaction (PCR). For the 40 mg/kg **A22** group, the transcriptions of BCL-2 and BAX were significantly different from those of the FA-induced 24/48 h group. Apoptosis occurs through signal transduction pathways, which can be mediated by various factors. In order to determine the pathway by which **A22** regulates cell apoptosis, a Western blot was used to study the related protein expressions. As shown in Figure 6C, in FA-induced 24/48 h groups, the levels of pro-apoptosis-related proteins BAX, Cyto-C, cleaved caspase 9, and cleaved caspase 3 were remarkably enhanced, while the levels of anti-apoptosis-related proteins BCL-2, caspase 9, and caspase 3 were significantly decreased. As expected, after treatment with **A22**, the levels of pro-apoptosis-related proteins were remarkably decreased, while the levels of anti-apoptosis-related proteins were significantly increased in dose-dependent manners. The above results showed that **A22** up-regulated BCL-2 expression levels and ameliorated apoptosis of renal tubule cells in FA-induced mouse models through an intrinsic apoptosis pathway.

### 2.6. **A22** Could Alleviate FA-Induced Renal Oxidative Stress and Lipid Peroxidation Status in Mice Model

**Figure 7:**
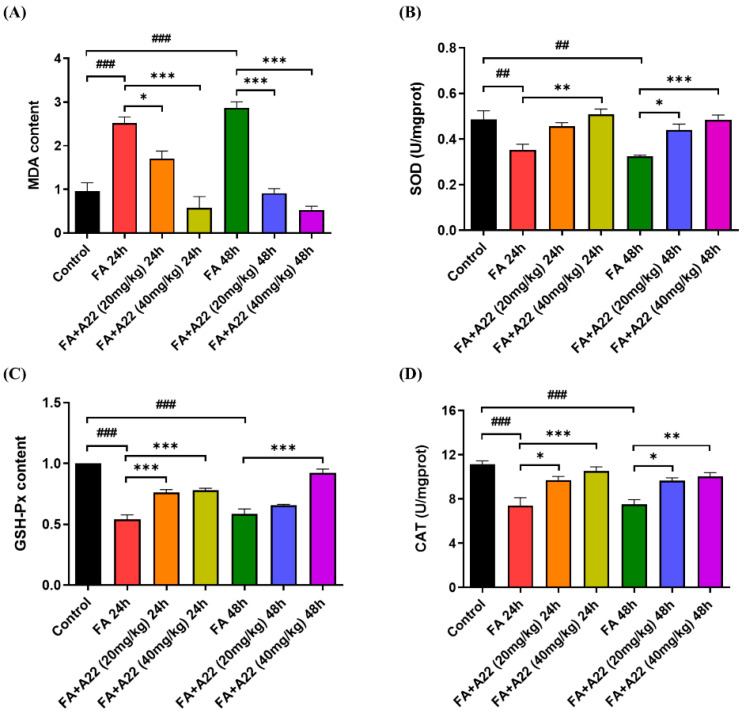
Effect of **A22** on kidney oxidative stress and lipid peroxidation status in acute kidney injury (AKI) mice. Some important indicators, including (**A**) superoxide dismutase (SOD), (**B**) malondialdehyde (MDA), (**C**) glutathione peroxidase (GSH-Px), and (**D**) cathepsin (CAT), were determined. Data were statistically analyzed as means ± SEM, with each circle or column indicating one group (N ≥ 3 mice/group). ## *p* < 0.01, vs. Control group; ### *p* < 0.001, vs. Control group; * *p* < 0.05, vs. FA 24/48 h group; ** *p* < 0.01, vs. FA 24/48 h group; *** *p* < 0.001, vs. FA 24/48 h group.

Oxidative stress plays an important role in AKI pathogenesis. The main pathogenic mechanism is an imbalance between oxidation and antioxidation in the body, which generates a large number of oxidation products and ultimately leads to oxidative stress injury [32,33]. Malondialdehyde (MDA) is known as a peroxidation product of membrane lipids in the organism, which is often used as an important indicator of oxidative damage to cells. Glutathione peroxidase (GSH-Px) is a peroxide-disintegrating enzyme widely present in the organism that reduces intracellular toxic peroxides through its own conversion, eliminating free radical damage and thus reducing the damage to peroxides in the organism [34]. Superoxide dismutase (SOD) is a metal enzyme that can catalyze the dismutation of superoxide anions, scavenge O^2−^, and repair damaged cells. Cathepsin (CAT) is a broadly distributed enzymatic scavenger that can break down H_2_O_2_ and protect cells from oxidative damage. To determine whether **A22** could ameliorate the kidney oxidative stress injury induced by the FA, we measured the kidney levels of the above oxidative stress-related parameters. As shown in Figure 7A–D, MDA (*p* < 0.001) was noticeably increased in the model group (FA-induced for 24/48 h) compared with that in the control group, while SOD (*p* < 0.01, *p* < 0.01), GSH-Px (*p* < 0.001, *p* < 0.001), and CAT (*p* < 0.001, *p* < 0.001) were markedly decreased. These data showed that FA aggravated lipid peroxidation and caused oxidative damage in kidney cells. Upon treatment of mice with different doses of **A22**, the intracellular levels of MDA were significantly reduced (*p* < 0.001, *p* < 0.05 and *p* < 0.001, *p* < 0.001), while SOD (*p* < 0.01 and *p* < 0.05, *p* < 0.001), GSH-Px (*p* < 0.001, *p* < 0.001 and *p* < 0.001) and CAT (*p* < 0.001, *p* < 0.05 and *p* < 0.01, *p* < 0.05) were markedly increased in dose-dependent manners. The above results indicated that **A22** could enhance the activity of GSH-Px, SOD, and CAT while reducing the kidney content of MDA, which could improve lipid peroxidation status and further protect the kidney from FA-induced oxidative stress damage.

### 2.7. **A22** Could Alleviate Endoplasmic Reticulum Stress (ERS) and Inflammatory Reaction in FA-Induced Mice Model

**Figure 8:**
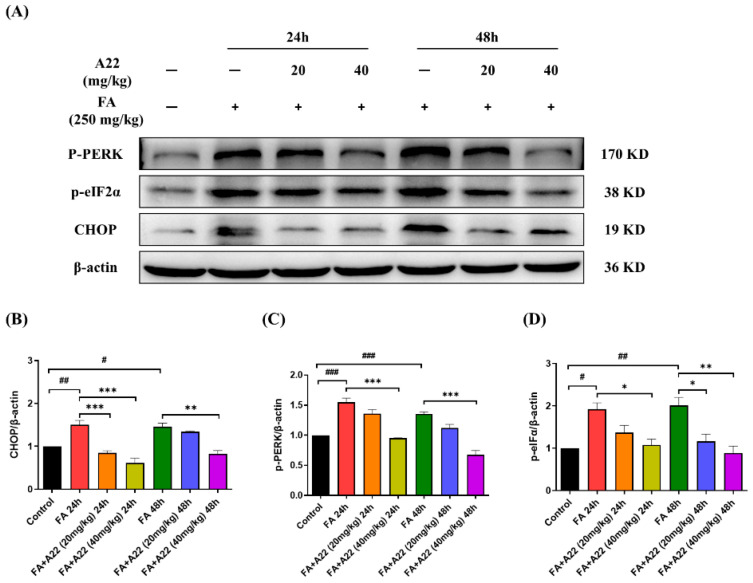
Effect of **A22** on endoplasmic reticulum stress (ERS) in folic acid (FA)-induced mice model. (**A**) Effect of **A22** on ERS. The unfolded protein response (UPR) proteins (PRKR-like endoplasmic reticulum kinase, P-PERK; phosphorylated eukaryotic translation initiation factor 2α, p-elF2α; and C/EBP homologous protein, CHOP) were analyzed by using Western Blot and corresponding histograms (**B**–**D**). Data were statistically analyzed as means ± SEM, with each circle or column indicating one group (N ≥ 3 mice/group). # *p* < 0.05, vs. Control group; ## *p* < 0.01, vs. Control group; ### *p* < 0.001, vs. Control group; * *p* < 0.05, vs. FA 24/48 h group; ** *p* < 0.01, vs. FA 24/48 h group; *** *p* < 0.001, vs. FA 24/48 h group.

It has been reported that increased renal tubular epithelial cell apoptosis plays a pivotal role in contributing to inflammation of AKI [35]. Many factors could lead to renal tubular epithelial cell apoptosis, including unresolved ERS [36] and inflammatory reactions [37]. Many studies have demonstrated that acute kidney injury could lead to ERS while exacerbating apoptosis and inflammatory responses [38]. Initiation of the unfolded protein response (UPR) classical pathway increases ERS, and accumulation of UPR could activate apoptosis. Therefore, we assessed the related protein factors PRKR-like endoplasmic reticulum kinase (P-PERK) and eukaryotic translation initiation factor 2α (eIF2α), which can initiate the UPR and are used to assess the potential therapeutic effect of **A22** on ERS in renal cells. As shown in Figure 8, expression levels of P-PERK, phosphorylated eIF-2α (p-eIF2α), and C/EBP homologous protein (CHOP) were all increased in the FA-induced mouse model compared with controls, which were then reduced in dose-dependent manners upon treatment with **A22**. Our results indicated that **A22** had a significant effect on ERS, which could contribute to ameliorating cell apoptosis.

**Figure 9:**
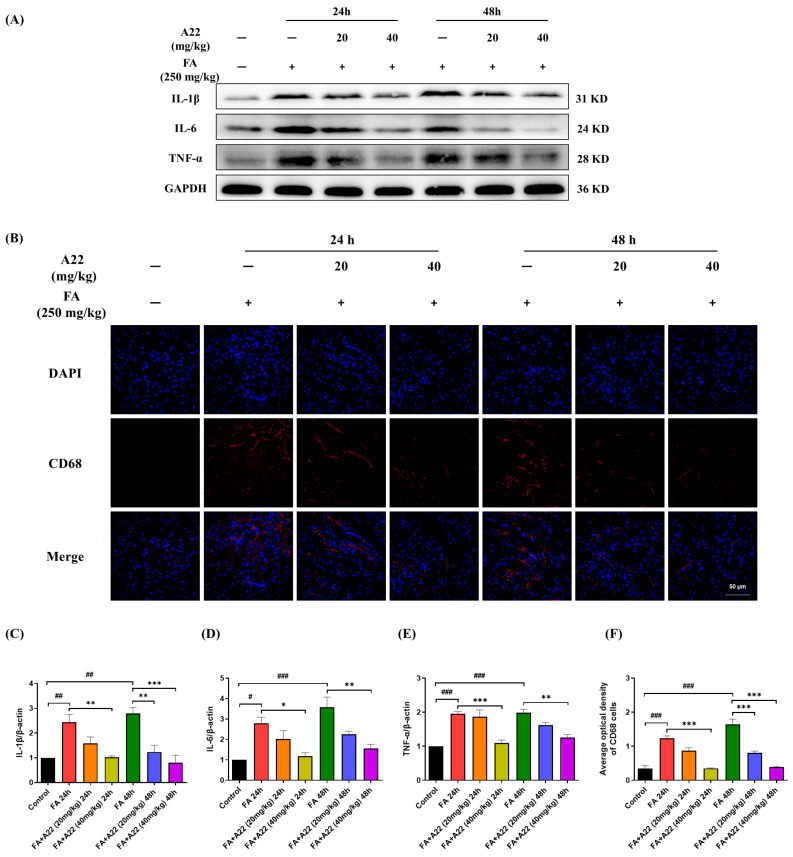
Effect of **A22** on inflammatory response in folic acid (FA)-induced mice model. (**A**) Effect of **A22** on expressions of inflammatory factors (tumor necrosis factor-α, TNF-α; interleukin-1 beta, IL-1β, and interleukin-6, IL-6) was analyzed by using Western Blot and corresponding histograms (**C**–**E**). (**B**) The infiltration density of the cluster of differentiation 68 (CD68) cells and corresponding statistical plot (**F**). Magnification was 60×, and the scale bar was 20 μm. Data were statistically analyzed as means ± SEM, with each circle or column indicating one group (N ≥ 3 mice/group). # *p* < 0.05, vs. Control group; ## *p* < 0.01, vs. Control group; ### *p* < 0.001, vs. Control group; * *p* < 0.05, vs. FA 24/48 h group; ** *p* < 0.01, vs. FA 24/48 h group; *** *p* < 0.001, vs. FA 24/48 h group.

It has been reported that during AKI, even when serum urea nitrogen (Sun) and serum creatinine (Scr) return to normal levels, the renal cellular inflammatory response could still exacerbate apoptosis, and inflammation continues to lead to renal cell loss, tubular cell damage, and renal imbalance [39]. Therefore, we studied whether **A22** could have an anti-inflammatory effect on FA-induced 24/48 h mouse kidneys. Our results showed that **A22** could significantly inhibit inflammatory cell aggregation in a mouse model of FA-induced AKI, analyzed against macrophage cluster of differentiation 68 (CD68) with the specific antigen, with the results as shown in Figure 9. **A22** could decrease expressions of inflammatory cytokines interleukin-1 beta (IL-1β), tumor necrosis factor-α (TNF-α), and interleukin-6 (IL-6) of the kidney in dose-dependent manners, as shown in Figure 9. These results suggested that **A22** was effective in attenuating not only FA-induced apoptosis of renal tubular epithelial cells but also pathological changes such as ERS, inflammatory response, fibrosis, and kidney damage.

## 3. Discussion

Thus far, diuretic drugs (to control volume overload) and renal replacement therapy are the main clinical treatments for AKI, and very limited clinical drug studies are available for AKI treatment. It has been shown that activation of caspases [40] and apoptosis of renal cells induced by BCL-2 family proteins play important roles in the activation of AKI. Apoptotic renal tubular epithelial cells can stimulate inflammatory cells to produce inflammatory factors and cytokines in the progression of AKI. Oxidative stress injury [41], lipid metabolism-associated [42], and endoplasmic reticulum stress [36] can accelerate the progress of AKI patients. The complex pathogenesis of AKI involves a reprogramming network of multiple pathogenic factors. Therefore, by targeting the relevant pathogenic factors involved in the pathway, multiple synergistic efforts can be used to provide a new and more effective strategy for the treatment of AKI. The anti-apoptotic factor BCL-2 seems to be an ideal target, and the restoration or up-regulation of its expression can initiate the caspase cascade reaction and inhibit mitochondria-mediated apoptosis. It has been shown that BCL-2 expression is significantly low for the kidneys of AKI patients [43,44], suggesting that the pathological progression of AKI is negatively correlated with BCL-2 expression level.

In a previous study, after extensive examination, we discovered acridone derivative **A22** could bind to and stabilize *BCL-2* gene promoter i-motif and subsequently up-regulate the expression of anti-apoptotic factor BCL-2, effectively alleviating steatohepatitis. We further applied the above research to the AKI disease model caused by excessive apoptosis of kidney cells, and our results showed that **A22** had a promising therapeutic effect. **A22** had a selective effect on intracellular gene expression, which up-regulated BCL-2 expression without significant effect on other gene expressions. It was verified through a knockout experiment that **A22** reduced renal tubular epithelial cell apoptosis by stabilizing the *BCL-2* gene promoter i-motif, resulting in up-regulation of the anti-apoptotic factor BCL-2 in the AKI model. **A22** could significantly improve the oxidative stress injury and pathological characteristics of animal models after reducing renal tubular epithelial cell apoptosis. Compared with other complex methods for up-regulating gene expressions, this strategy of targeting the gene promoter i-motif through small molecule binding is simpler and more practical. Significantly, *BCL-2*, as one of the most highly regarded genes in apoptosis research, plays an important role in the endogenous pathway regulating apoptosis. Our results showed that selective up-regulation of BCL-2 expression in renal cells could improve mitochondrial function while maintaining the mitochondrial number and blocking the caspase cascade triggered by apoptotic vesicles. In summary, **A22** could effectively restore renal function by inhibiting renal cell apoptosis, counteracting the occurrence of pathological changes in AKI, and attenuating acute kidney injury.

The down-regulation of BCL-2 expression has been observed in the kidneys of patients with AKI, indicating a significant involvement of BCL-2 in the apoptosis of renal tubular epithelial cells during AKI. In this study, **A22** showed evident anti-apoptotic effects in various aspects. Through up-regulating BCL-2 expression, **A22** could maintain the number and function of mitochondria, prevent the release of pro-apoptotic factors (e.g., cytochrome c) from the mitochondria into the cytosol, and block the activation of caspase cascades through inhibition of csapase3 and csapase9, which indicated its possible multi-functional anti-apoptosis mechanism. The AKI process is multifactorial and complex, involving the interplay among apoptosis, inflammation, oxidative stress, ER stress, and fibrosis. Since **A22** could play an anti-apoptotic effect in AKI, we investigated whether it could ameliorate pathological changes in the AKI process. We found that **A22** could effectively reduce the level of Bun and Scr, thereby attenuating kidney injury and mitigating morphological damage to the kidney tissues. The kidneys of mice induced by FA exhibited an elevated accumulation of inflammatory factors, characterized by the up-regulation of these factors and the gathering and accumulation of mononuclear macrophages. **A22** could effectively reduce the expressions of inflammatory factors and attenuate inflammatory cell gathering. Fibrosis at an early stage of AKI was also reversed in FA-induced 24/48 h mice upon treatment with **A22,** as shown in Figure S2. In addition, oxidative stress injury and lipid metabolism disorder also showed dose-dependent improvement upon treatment with **A22**.

Our present study showed that **A22** could become a promising lead compound for further research and development for AKI treatment. Our employed model focused on its short-term effect on AKI, while its long-term efficacy and safety, particularly regarding the regeneration of renal tubular epithelial cells and the potential risk of cancer, as well as its possible off-target effects, require further investigation before clinical application. It should be noted that the observations made in our research are short-term in nature, without any observation of regeneration. On the other hand, clinical AKI is pathologically characterized by injury and death of tubular epithelial cells. After the initial injury, surviving tubular epithelial cells undergo dedifferentiation for proliferation and kidney repair. For this reason, it is probable that initial apoptosis may be a necessary mechanism for the elimination of damaged cells so that only undamaged cells undergo dedifferentiation and only such cells are the basis for subsequent regeneration lasting weeks or months. In such cases, the occurrence of apoptosis could be a beneficial phenomenon, while the limitation of apoptosis could impair regeneration and lead to the development of renal cancer. On the other hand, **A22** was effective in attenuating not only FA-induced apoptosis of renal tubular epithelial cells but also pathological changes such as ERS, inflammatory response, fibrosis, and kidney damage, which could be beneficial for the regeneration of renal tubular epithelial cells. The answer to this debate can only be obtained after its long-term efficacy and safety study. It is also likely that **A22** affects other apoptotic pathways, which could also be investigated in the future. Besides hnRNP LL, other transcription factors might also be involved in the mechanism, which could be studied in the future. Nevertheless, our present study provided a new possible solution for AKI treatment, which shed light on further research and development of **A22** as a novel anti-apoptotic lead compound for in-depth investigation.

## 4. Material and Methods

### 4.1. Cell Culture and Cell Counting Kit-8 (CCK-8) Assays

HK-2 cells were immortalized by transduction with human papilloma virus 16 (HPV-16) E6/E7 genes, and the cells were obtained from Shanghai Sixin Biotechnology Co., Ltd. (Shanghai, China). HK-2 cells were cultured in an incubator with 5% CO_2_ at 37 °C with 10% fetal bovine serum (Bio-Channel, Nanjing, China) and 1% penicillin/streptomycin (Thermo, Guangzhou, China).

HK-2 cells were seeded in a 96-well plate (6000 cells/well) in 100 μL medium and cultured overnight. After the cells adhered to the well, different doses of folic acid (0–3 mg/mL FA, BIDE, Shanghai, China) and **A22** (0–80 μM) were added, and the cells were continuously incubated for 24 h. Then, 10 μL CCK-8 solutions (Solarbio, Beijing, China) were added to each well, and the mixtures were continuously incubated for another 4 h. The experiments were carried out in triplicate for all drug doses. A Bio-Rad 680 microplate reader was utilized to measure the absorbance at 450 nm (CCK-8 assay), and the relative survival ratio of cells was determined through a calculation based on the following formula:


$$Relative cell survival ratio\;(\%)=\frac{{A450}_{Sample}-{A450}_{Blank}}{{A450}_{Control}-{A450}_{Blank}}\times 100$$

The absorbance of the blank, sample, and control at 450 nm was expressed as A450_Blank_, A450_Sample_, and A450_Control_, respectively.

### 4.2. siRNA Interference Silencing Experiment

HK-2 cells were cultured in 96 or 6-well plates and ready for transfection after the cells covered 70–80% of the plate. siRNA fragments were obtained from Guangzhou Ruibo Biotechnology Co., Ltd (Guangzhou, China). with sequences as shown in Table S1. After screening, the data were obtained as shown in Figure S1, and the best siRNA fragment of the hnRNP LL gene was diluted to the final concentration of 50 nM. In the meantime, lip3000 (Thermo, Guangzhou, China) was diluted with OPTI-MEM medium (Thermo, Guangzhou, China), which was mixed with the above fragment in a 1:1 ratio and then transferred into the culture dish for transfection. The compounds at different concentrations and FA were added, and then the samples were collected for analysis.

### 4.3. FITC Annexin V/PI Apoptosis Detection

FITC Annexin V/PI apoptosis detection was performed with the FITC Annexin V/PI Apoptosis Detection Kit (Muti Sciences, Hangzhou, China). Briefly, after treatment of FA-induced cells or FA-induced knockout cells with different doses of **A22** (20, 40 μM), then the cells were washed 3 times with 1×PBS (Servicebio, Guangzhou, China), digested, and resuspended in 500 μL of freshly prepared binding buffer. The cells were co-incubated with Annexin FITC V (10 μL) and PI (5 μL) for 10 min at room temperature in a darkroom. The fluorescence was measured using flow cytometry (CytoFLEX S, Beijing, China), and the collected data were analyzed using CytExpert2.4 software.

### 4.4. ROS and Mitochondrial Function Measurement

HK-2 cells were plated on 15 mm confocal plates. The intracellular levels of ROS were assessed using a test kit containing 2′,7′-dichlorofluorescein diacetate (DCF, Beyotime, Haimen, Jiangsu, China), following the guidelines provided by the manufacturer. After 24 h treatment with **A22** (20, 40 μM) in a 3 mg/mL FA-induced model, the cells were washed three times with cold 1×PBS to remove remaining DCF and 4′,6-diamidino-2-phenylindole (DAPI, Beyotime, Haimen, Jiangsu, China). The ROS generation was analyzed by using fluorescence microscopy (488 nm filter, Olympus, Tokyo, Japan).

Mitochondrial function was evaluated using a JC-1 fluorescence probe (Meilunbio, Dalian, China) and Mito tracker deep red staining. Cells were prepared in the same way as before. After 24 h treatment with **A22** (20, 40 μM), the cells were incubated with DAPI and Mito tracker deep red (100 nM, Sigma, Shanghai, China) or JC-1 fluorescence probe for the time specified by the kit at 37 °C in the dark. Then, the cells were washed three times with PBS to remove the remaining Mito tracker deep red, JC-1, and DAPI. All the samples were photographed using FV3000 (Olympus, Tokyo, Japan). Fluorescence detection was carried out by using EVOS FL Auto (Thermo, Guangzhou, China). Image J 1.8.0 software (Bio-Rad Laboratories, Hercules, CA, USA) was used to count the proportion of positive fluorescent areas.

### 4.5. Animal Experiment

SPF-grade male C57BL/6 mice at the age of 6 weeks, with a weight of 18–22 g, were bred in the laboratory at the Animal Center of Sun Yat-sen University (Guangzhou, China) for experimental use. The procedures for animal feeding and experimentation were in accordance with legal requirements and national guidelines for the care and maintenance of laboratory animals, which were approved by the Animal Protection and Use Committee of Sun Yat-sen University (Approval No. SYSU-IACUC-2021-000004). Approval date: 7 January 2021. Effective date: 7 January 2021 to present. Animals were housed in an SPF room with constant temperature at 21–23 °C on a 12 h dark/light cycle. After 1 week of adaptive feeding, the mice were divided into seven groups (a total of 42 mice, 6 per group), with treatment as shown in Table 1.

### 4.6. Reverse Transcription and Real-Time PCR

Total RNA was extracted from HK-2 cells or excised kidney tissues, followed by purification. Fresh tissue or cell samples were washed 2–3 times with 1×PBS, and samples were homogenized after adding a homogenizing solution. Following the addition of chloroform, the solution was mixed and left to stand at room temperature for 5 min before centrifugation. The transparent upper aqueous layer containing RNA was collected, followed by the addition of isopropanol to precipitate RNA from the aqueous layer. The solution was centrifuged, and ethanol/water was used to clean the RNA precipitate, which was finally dissolved in DEPC water to give the fresh RNA sample. The cDNA was then prepared following PCR Kit instructions (Takara, Beijing, China).

PCR was carried out using 2×RealStar SYBR Mixture (GenStar, Guangzhou, China). The data were calculated based on the threshold cycle values (Ct) and relative mRNA expression levels. The primers were all synthesized by Bioengineering Biotechnology (Guangzhou, China), with their sequences as shown in Table S1. Actin or GAPDH was used as a control, and relative mRNA levels were normalized with the control group.

### 4.7. Assessment of Blood Urea Nitrogen (Bun) and Serum Creatinine (Scr)

For the measurement of renal function, the levels of Bun and Scr were examined by using ELISA kits (Jiancheng Biotech, Nanjing, China). The AKI model was successfully established when the Scr of the FA 24/48 h groups rose up to 2-fold of their control littermates.

### 4.8. Biochemical Parameters Analysis

Kidney tissue protein extraction was performed using a previously reported method, and the BCA Protein Assay Kit (Beyotime, Haimen, Jiangsu, China) was used to measure the protein content of each sample according to the instructions. The contents of MDA, SOD, CAT, and GSH-Px were determined using ELISA kits (Beyotime, Haimen, Jiangsu, China).

### 4.9. Renal Histopathological Examination

Kidney tissues were fixed in 4% paraformaldehyde, embedded in paraffin, and sectioned. It is worth noting that the sections need to be dewaxed and repaired prior to staining, and the kidney sections were stained with PAS, HE, and Masson staining (Servicebio, Guangzhou, China). The sections were observed under optical microscopy (EVOS FL Auto, Carlsbad, CA, USA) at magnifications of ×200 or ×400. Tubular injury was characterized by swelling of tubular epithelial cells, disruption of the brush border, degeneration with vacuoles, necrosis in tubules, and formation of casts. To assess the extent of renal damage, 10 fields were randomly selected at a magnification of ×200 from each sample. The Paller method was used to evaluate the histopathological changes, and tissue damages of renal tubules were scored on a scale of 0–4.

### 4.10. Immunohistochemical Staining and TUNEL Assay

The treated slices of kidney tissues were analyzed by using immunohistochemistry to assess the expression levels of BCL-2 and CD68 in each group. The kidney tissue sections were incubated with 3% hydrogen peroxide for 15 min at room temperature to block endogenous peroxidase activity. The sections were blocked with 5% goat serum and then incubated with primary antibodies against BCL-2 (1:500; Servicebio, Guangzhou, China) and CD68 (1:500; Servicebio, Guangzhou, China) overnight at 4 °C. Then, the sections were washed with PBS three times and incubated with biotinylated secondary antibodies at room temperature for 40–60 min. At the end of the secondary antibody incubation, the sections were washed three times with 1×PBS, and cell staining was performed through the addition of DAPI, and the sections were finally sealed. The collected mouse kidney samples were washed with 1×PBS, fixed in 10% paraformaldehyde, embedded in paraffin wax, and then sectioned. After dewaxing and restoration, all the sections were incubated with 20 mg/mL proteinase K (Servicebio, Guangzhou, China) for 15 min at room temperature to remove protein. The apoptosis detection kit from Roche (Servicebio, Guangzhou, China) was used for the experiment, and TUNEL staining was performed according to the instruction manual. The nuclei were localized using DAPI (Servicebio, Guangzhou, China), and the slices were sealed and placed under an optical microscope (Olympus, Tokyo, Japan) at a magnification of 40–60× to observe the TUNEL-positive cells. TUNEL-labeled cells, and the levels of BCL-2 and CD68 in each group were calculated with Image J 1.8.0 software.

### 4.11. Protein Extracts and Western Blotting

Fresh cells or kidney tissue were washed with 1×PBS and pre-cooled RIPA (Beyotime, Chengdu, China), and protease inhibitors were added and lysed on ice for 30 min. Then, extracted proteins were quantified and denatured, which were separated by using 10% SDS-PAGE (Servicebio, Guangzhou, China) and electro-transferred to a 0.22 um PVDF membrane (Millipore, Guangzhou, China). The membranes were blocked with 5% bovine serum albumin (BSA, Beyotime, Haimen, Jiangsu, China) in TBST (0.1%, Servicebio, Guangzhou, China) for 1 h at room temperature and then incubated with different primary antibodies (as shown in Supporting Materials) at 1:1000 dilution in 5% BSA at 4 °C overnight on the rotating shaker. The membranes were washed with 1 × TBST for 3 × 15 min to remove unbound antibodies and then incubated with a secondary antibody (Cell Signaling Technology, Cat#7076 from mouse and Cat#7074 from rabbit, Shanghai, China). The protein bands were visualized with an ECL Luminous Liquid (New Cell Molecular, Suzhou, China). The signal values for each target protein were counted using Image J 1.8.0 software (Bio-Rad Laboratories, Hercules, CA, USA) and normalized to the control group. Data were expressed as the mean ± SEM. Data between the two groups were analyzed with one-way ANOVA or unpaired Student’s *t*-tests using GraphPad9.0 Prism (GraphPad9.0 Software Inc., Solana Beach, CA, USA). A * *p*-value of ≤ 0.05 is considered statistically significant.

## Figures and Tables

**Table 1:** The groups of mice and their treatment in animal experiments.

Group	Administration
Control FA 24 h	0.3 M NaHCO_3_, i.p. FA (250 mg/kg, dissolved in 0.3 M NaHCO_3_, i.p.)
FA 24 h + **A22** (20 mg/kg)	FA (250 mg/kg, dissolved in 0.3 M NaHCO_3_, i.p.) and **A22** (20 mg/kg, i.p.)
FA 24 h + **A22** (40 mg/kg)	FA (250 mg/kg, dissolved in 0.3 M NaHCO_3_, i.p.) and **A22** (40 mg/kg, i.p.)
FA 48 h	FA (250 mg/kg, dissolved in 0.3 M NaHCO_3_, i.p.)
FA 48 h + **A22** (20 mg/kg)	FA (250 mg/kg, dissolved in 0.3 M NaHCO_3_, i.p.) and **A22** (20 mg/kg, i.p.)
FA 48 h + **A22** (40 mg/kg)	FA (250 mg/kg, dissolved in 0.3 M NaHCO_3_, ip) and **A22** (40 mg/kg, i.p.)

FA means folic acid. i.p. means intraperitoneal injection.

## Data Availability

The data presented in this study are available in both the article and Supplementary Material.

## Supplementary Materials

The following supporting information can be downloaded at: https://www.mdpi.com/article/10.3390/ijms252212028/s1.
