# Supplementary material for: New Therapeutic Method for Alleviating Damage of Acute Kidney Injury Through BCL-2 Gene Promoter I-Motif
Source: Int J Mol Sci. 2024 Nov 8;25(22):12028. doi: 10.3390/ijms252212028 (PMC11593768; doi:10.3390/ijms252212028)
Supplement: Supplementary file 1 [file ijms-25-12028-s001.zip › ijms-3274134-supplementary.pdf]

International Journal of *Molecular Sciences*

MDPI

Supplementary Material for

New therapeutic method for alleviating damage of acute kidney injury through *BCL-2* gene promoter i-motif

Dongsheng Ji[1], Jiahui Zhang[1], Jihai Liang[1], Jing Wang[1], Xiaoya Li[1,2,*], Zhi-Shu Huang[1], and Ding Li[1,*]

[1] School of Pharmaceutical Sciences, Sun Yat-sen University, Guangzhou University City, Guangzhou 510006, P. R. China

[2] Department of Nephrology, Nanfang Hospital, Southern Medical University, No. 1838, Guangzhou Avenue North, Baiyun District, Guangzhou, Guangdong, China

* Corresponding author:

Ding Li; Tel: 8620 3994 3058; E-mail: liding@mail.sysu.edu.cn

Xiaoya Li; Tel: 8620 3994 3070; E-mail: lixiaoya0908@126.com

Table of Contents

Table S1. Primer sequence information S3
Table S2. The qPCR raw data from real-time RT-PCR experiment for cells and animal samples S4
Figure S1. Screening of siRNA fragments for the best silencing effect and subsequent Western blot experiment S6
Figure S2. MASSON staining of mice kidney tissue showed that **A22** improved renal fibrosis in mice model S7
Figure S3. Three repetitions of the strips used to count the gray values in Figure 1 S9
Figure S4. Three repetitions of the strips used to count the gray values in Figure 3 S9
Figure S5. Three repetitions of the strips used to count the gray values in Figure 4 S10
Figure S6. Three repetitions of the strips used to count the gray values in Figure 6 S10
Figure S7. Three repetitions of the strips used to count the gray values in Figure 8 S11
Figure S8. Three repetitions of the strips used to count the gray values in Figure 9 S11
MATERIAL AND METHODS S12

Table S1. Primer sequence information

Spics	Genes	Forward primer (5′-3′)	Reverse primer (5′-3′)
	BCL-2	TGTTGTTCAAACGGATTCA	GGCTGGGCACATTTACTGTT
	BAX	AGCGACTGATGTCCCTGTCT	CTCAGCCCATCTTCTTCCAG
	β-actin	CTGGAACGGTGAAGGTGAA	AAGGGACTTCTGTAACAACGA
human	siRNA 1	GCAAAGUGCAACGUAUUGUUATT	UAACAAUACGUUGCACUUUGCTT
	siRNA 2	CCUUCUUCGUUUAGACAUGAUTT	AUCAUGUCUAAACGAAGAAGGTT
	siRNA 3	GCACUGAAUCACUAUCAGAUATT	UAUCUGAUAGUGAUUCAGUGCTT
	BCL-2	GTACCTGAACCGGCATCTG	GGGGCCATATAGTTCCACAA
mice	BAX	TAGCAAACTGGTGCTCAAGG	TCTTGGATCCAGACAAGCAG
	β-actin	GACCTCTATGCCAACACAGTGC	GTACTCCTGCTTGCTGATCCAC

Table S2. The qPCR raw data from real-time RT-PCR experiment for cells and animal samples

Experiment 1	Ct		a-ΔΔCt a	bF = $2^{-\Delta\Delta Ct}$
	β-actin	BCL-2		
Control	13.26	25.28	0	1
3 mg/ml FA	13.45	27.84	0.49	0.71
3 mg/ml FA +20 μM **A22**	13.86	25.02	0.013	0.99
3 mg/ml FA + 40 μM **A22**	12.82	25.26	-0.43	1.35

Experiment 2	Ct		a-ΔΔCt a	bF = $2^{-\Delta\Delta Ct}$
	β-actin	hnRNP LL		
Control	25.14	18.69	0	1
siRNA1	26.40	21.52	1.39	0.34
siRNA2	28.31	22.51	0.47	0.63
siRNA3	26.12	20.31	0.45	0.64
	β-actin	BCL-2	a-ΔΔCt a	bF = $2^{-\Delta\Delta Ct}$
Control	25.13	21.38	0	1
siRNA1	26.39	24.27	1.07	1.0
siRNA2	28.31	24.76	-0.35	0.81
siRNA3	25.45	21.26	-0.49	0.89
	β-actin	BAX	a-ΔΔCt a	bF = $2^{-\Delta\Delta Ct}$
Control	25.23	20.8	0	1
siRNA1	26.40	21.69	0.05	1.21
siRNA2	28.31	23.6	0.05	1.21
siRNA3	26.12	21.58	0.33	0.93

Experiment 3	Ct		a-ΔΔCt a	bF = $2^{-\Delta\Delta Ct}$
	β-actin	BCL-2		
Control	15.45	31.635	0	1
FA 24h	15.43	32.33	0.63	0.64
FA 24h + 20 mg/kg **A22**	15.97	31.35	0.62	0.65
FA 24h + 40 mg/kg **A22**	16.49	30.26	0.32	0.80
FA 48h	15.73	28.535	1.04	0.49
FA 48h + 20 mg/kg **A22**	15.88	31.35	0.65	0.63

FA 48h + 40 mg/kg **A22**	15.73	30.26	0.4	0.75
	β-actin	BAX	[a]-ΔΔCt[a]	[b]F = $2^{-\Delta\Delta Ct}$
Control	16.16	20.45	0	1
FA 24h	15.52	18.91	-0.68	1.60
FA 24h + 20 mg/kg **A22**	15.99	19.88	-0.26	1.20
FA 24h + 40 mg/kg **A22**	15.53	19.54	-0.103	1.07
FA 48h	16.60	19.56	-1.17	2.25
FA 48h + 20 mg/kg **A22**	15.60	19.3	-0.42	1.34
FA 48h + 40 mg/kg **A22**	15.79	19.82	-0.1	1.07

[a] **-ΔΔCt** = [(Ct value of genes in treated sample - Ct value of genes in control) - (Ct value of GADPH in treated sample - Ct value of GADPH in control)]. [b] F: The relative expression fold.
Experiment 1: The Ct values of *BCL-2* in 3 mg/ml FA model with different concentration of **A22**
Experiment 2: HK-2 cells were treated with different fragments of siRNA. The Ct values of hnRNP LL, BCL-2 and BAX were obtained.
Experiment 3: The Ct values of *BCL-2* and Bax in FA-induced and **A22** administration group of mice kidney.

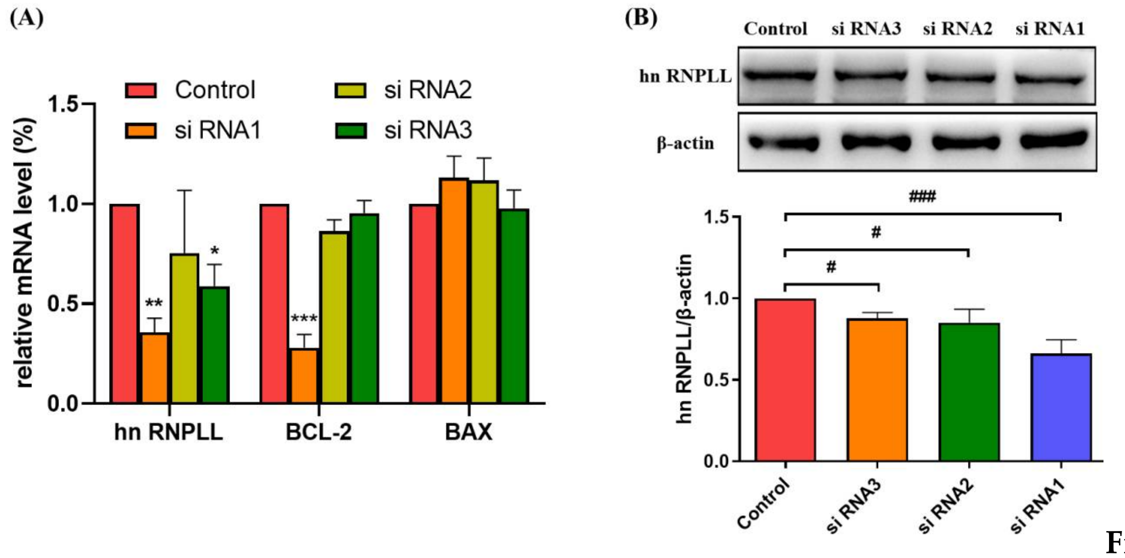

Figure S1. Screening of siRNA fragments for the best silencing effect and subsequent Western blot experiment. (A) The mRNA levels of hnRNP LL, BCL-2 and BAX. (*p<0.05, **p<0.01 and ***p<0.001 vs. Control group). (B) The changes of hnRNP LL in HK-2 cells and corresponding histogram after siRNA silencing with oligomers of different sequences. The extracted proteins from the cells were immunoblotted with specific antibodies, and quantified based on the loading control of ACTIN (###p<0.001, vs. Control group). These data showed that siRNA1 had a better silencing effect compared to other siRNA fragments, so the fragment of siRNA1 was selected for target verification.

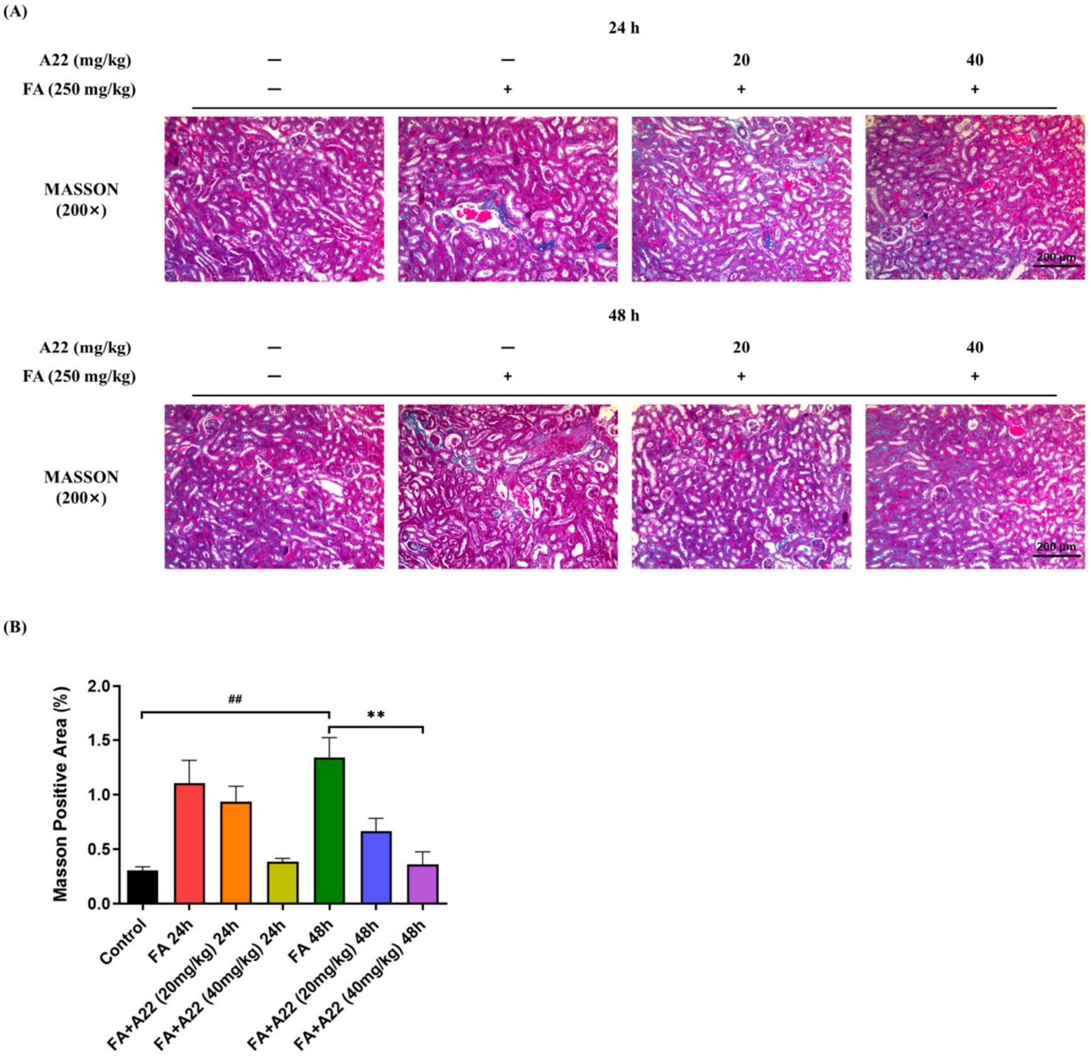

Figure S2. MASSON staining of mice kidney tissue showed that **A22** improved renal fibrosis in mice model. (A) Masson staining of the kidney tissues in mice (Magnification was 200x, and scale bar was 200 μm). As to kidney fibrosis, compared with control groups, the interstitial and perivascular collagen depositions were also obviously found in kidneys of FA groups. **A22** could alleviate collagen deposition of FA induced kidney injury tissues. Image J software was used to count the collagen deposition area, and **A22** could reduce collagen deposition area in a dose-dependent manner. Data were statistically analyzed as shown in (B) as mean ± SEM, with each column indicating one group. ##p<0.01, vs. Control group; **p<0.01, vs. FA 48h group.

MATERIAL AND METHODS

RNA extraction and qRT-PCR

Total RNA from cells and mice kidney was isolated by using RNAiso Plus (Takara, Cat# 9109, Japan). After homogenizing the sample, chloroform was added, and the homogenate was allowed to separate into a clear upper aqueous layer (containing RNA), an interface, and a red lower organic layer (containing the DNA and proteins). RNA was precipitated from the aqueous layer with isopropanol. DNA was precipitated from the aqueous/organic interface with ethanol. cDNA was synthesized with a One-Step RT-PCR Kit (Takara, Cat# 639503, Japan).

Quantitative real-time polymerase chain reaction (PCR) was carried out using 2×RealStar SYBR Mixture (GenStar, Cat# A301-10, Guangzhou, China). The results were analyzed on a LightCycler480 II real-time PCR system (ROCHE, USA) using the 2-ΔΔCt method. Primers were synthesized by Sangon Biotec (Guangzhou, China) with sequences listed as shown in Table S1. Actin was used as a loading control and relative mRNA levels were normalized to Actin.

Western blotting

Cell samples were lysed in cold RIPA extraction buffer (Beyotime, Cat# P0013C, Chengdu, China) with an addition of protease inhibitors (Roche, Cat# 4693006001, Guangzhou, China). The extracted proteins were separated by using SDS-PAGE and transferred to a polyvinylidene difluoride membrane (Millipore, Guangzhou). After blocking with TBS/T (0.1%) containing 5% bovine serum albumin (BSA) for 25-30 min at room temperature, the membrane was incubated with different primary antibodies of hnRNP LL (Affinity Biosciences OH, Cat# AF6139, USA), by 1:1,000 dilution in 5% bovine serum albumin at 4 °C overnight. The membrane was washed with TBS/T for 4 x 10 min to remove unbound antibodies, and then incubated with HRP-conjugated secondary antibodies (Cell Signaling Technology, Cat# 7076 from mouse and Cat# 7074 from rabbit, China). Protein bands were visualized with an ECL kit (Millipore, Cat# 64-201BP, China). Densitometry analysis was performed using Image J Software (Bio-Rad Laboratories, CA, USA) relative to the loading control.

The types of primary antibodies were BCL-2 (affinity biosciences OH, Cat# AF6139, USA), BAX (affinity biosciences OH, Cat# AF0120, USA), Cyto-c (affinity biosciences OH, Cat# AF0146, USA), Caspase9 (affinity biosciences OH, Cat# AF6348, USA), cleaved-Caspase9 (affinity biosciences OH, Cat# AF5240, USA), Caspase3 (affinity biosciences OH, Cat# AF6311, USA), cleaved-Caspase3 (affinity biosciences OH, Cat# AF7022, USA), P-PERK (Cell Signaling Technology, Cat# 3192S, USA), P-eIF-2α (Cell Signaling Technology, Cat# 3298T, USA), CHOP (Cell Signaling Technology, Cat# 2895T, USA), IL-6 (Cell Signaling

Technology Cat# 12153, USA), TNF-α (Cell Signaling Technology Cat# 6945, USA), IL-1β (Cell Signaling Technology Cat# 6956, USA)
